# Habitual chocolate consumption and the risk of incident heart failure among healthy men and women

**DOI:** 10.1016/j.numecd.2016.01.003

**Published:** 2016-08

**Authors:** C.S. Kwok, Y.K. Loke, A.A. Welch, R.N. Luben, M.A.H. Lentjes, S.M. Boekholdt, R. Pfister, M.A. Mamas, N.J. Wareham, K.-T. Khaw, P.K. Myint

**Affiliations:** a School of Medicine, Medical Sciences & Nutrition, University of Aberdeen, Aberdeen, Scotland, United Kingdom; b Keele Cardiovascular Research Group, Institute for Science & Technology in Medicine, Keele University, Stoke-on-Trent, United Kingdom; c Department of Population Health & Primary Care, Norwich Medical School, University of East Anglia, Norwich, United Kingdom; d Department of Public Health & Primary Care, University of Cambridge, Cambridge, United Kingdom; e Department of Cardiology, Academic Medical Center, Amsterdam, The Netherlands; f Department III of Internal Medicine, Heart Centre of the University of Cologne, Cologne, Germany; g Medical Research Council Epidemiology Unit, Cambridge, United Kingdom

**Keywords:** Heart failure, Chocolate, Cocoa, Epidemiology, Meta-analysis

## Abstract

**Background:**

We aimed to examine the association between chocolate intake and the risk of incident heart failure in a UK general population. We conducted a systematic review and meta-analysis to quantify this association.

**Methods and results:**

We used data from a prospective population-based study, the European Prospective Investigation into Cancer (EPIC)-Norfolk cohort. Chocolate intake was quantified based on a food frequency questionnaire obtained at baseline (1993–1997) and incident heart failure was ascertained up to March 2009. We supplemented the primary data with a systematic review and meta-analysis of studies which evaluated risk of incident heart failure with chocolate consumption. A total of 20,922 participants (53% women; mean age 58 ± 9 years) were included of whom 1101 developed heart failure during the follow up (mean 12.5 ± 2.7 years, total person years 262,291 years). After adjusting for lifestyle and dietary factors, we found 19% relative reduction in heart failure incidence in the top (up to 100 g/d) compared to the bottom quintile of chocolate consumption (HR 0.81 95%CI 0.66–0.98) but the results were no longer significant after controlling for comorbidities (HR 0.87 95%CI 0.71–1.06). Additional adjustment for potential mediators did not attenuate the results further. We identified five relevant studies including the current study (N = 75,408). The pooled results showed non-significant 19% relative risk reduction of heart failure incidence with higher chocolate consumption (HR 0.81 95%CI 0.66–1.01).

**Conclusions:**

Our results suggest that higher chocolate intake is not associated with subsequent incident heart failure.

## Highlights

• Whether chocolate consumption increases or decreases the risk of heart failure remains controversial.
• Our results suggest that higher chocolate intake is not associated with subsequent incident heart failure.
• Our meta-analysis found consistent findings across all studies that chocolate consumption does not increase the risk of incident heart failure.
• Evidence suggests that chocolate consumption does not increase risk of heart failure at the general population level.

## Introduction

Cocoa, one of the main ingredients in plain chocolate, is an important dietary source of flavonoids which are believed to have cardiovascular benefits. Both observational and trial evidence have shown that chocolate consumption is associated with a reduction in blood pressure [1], [2], [3], [4]. This reduction in blood pressure is thought to be due to enhancement of endothelial nitric oxide production [5], [13] and inhibition of angiotensin converting enzyme [6], [7]. Flavonoids also increase antioxidant capacity and diminish the production of oxidative products in the plasma [8] and cocoa (one of the ingredients) has been shown to inhibit oxidation of low density lipoprotein (LDL) [9]. Moreover, the intake of chocolate and cocoa-based products increases high density lipoprotein (HDL) cholesterol [10], reduces inflammation [11], [12] and inhibits platelet aggregation [13]. Indeed, we have recently reported that habitual chocolate consumption is associated with lower cardiovascular risk [14]. These factors in combination may reduce the incidence of heart failure with higher chocolate consumption.

Existing observational studies evaluating the risk of heart failure with chocolate consumption show conflicting results and are not directly applicable to the general population. They were conducted in highly selective populations such as women who underwent mammography [15], older women who received calcium therapy [16], in those after myocardial infarction [17], and among male physicians [18]. To date, there has not been a study on the general population which evaluates the relationship between habitual chocolate intake and subsequent risk of heart failure.

In this paper, we report results from a large prospective UK population study, the European Prospective Investigation into Cancer-Norfolk (EPIC-Norfolk), and quantify the effect of chocolate consumption on heart failure incidence by conducting a systematic review and meta-analysis, including the current study.

## Methods

### EPIC-Norfolk cohort study

The study methods of the European Prospective Investigation into Cancer (EPIC)-Norfolk have been previously described in detail [19]. In brief, this is a prospective population study of 25,639 men and women, resident in Norfolk, United Kingdom (99.6% white Caucasian). A baseline survey was conducted between 1993 and 1997. Participants completed a health and lifestyle questionnaire and attended a health examination at their general practitioner's clinic where a non-fasting venous blood sample was taken and stored.

The data collection methods are described in Appendix 1. Measurements of height, weight, body mass index; laboratory methods; ascertainment of education status, social class, self-reported physical activity, smoking status, alcohol consumption, and identification of participants' co-morbid conditions using questionnaires have been described in previous studies [19], [20], [21], [22]. A food frequency questionnaire (FFQ) was used to assess overall diet in the past year [23], [24].

A one year recall of chocolate consumption was estimated using three questions from the FFQ and these questions were consumption of: “Chocolates singles or squares” (average portion size of 8 g), “Chocolate snack bars, e.g. Mars, Crunchie” (average portion size of 50 g) and “Cocoa, hot chocolate (cup)” (average portion size of 12 g powder weight). The amount of chocolate product eaten (grams/day) was derived from multiplying the frequency categories by the portion size. The sum of the weights of these food items consumed, rather than their flavonoid or cocoa content, formed the measure of exposure. Chocolate intake ranged from 0 to 348 g/d. We excluded participants with chocolate intake greater than 100 g/d (n = 68), since generalization of the results to such high quantities seemed unjustified. Equally, errors (i.e. outliers) relating to participant's poor comprehension of FFQs in general, or specifically the questions on chocolate products, were hereby minimized.

### Ascertainment of heart failure incidence

Hospital admissions for participants were identified using the participant's unique National Health Service number and linking this to the ENCORE (**E**ast **N**orfolk **CO**mmission **RE**cord) database. All participants were flagged for death certification at the UK Office of National Statistics, ascertaining vital status for the entire cohort. Incident heart failure cases were ascertained by using death certificate data and hospital record linkage using the “International Classification of Disease-10” (ICD10) code I50 with virtually complete follow up [22]. Heart failure ascertainment has been previous validated and reported [25]. We reported data with follow-up up to March 2009, an average of 12.5 years. The study was approved by the Norwich District Health Authority Ethics Committee. All participants provided signed informed consent.

### Statistical analysis

Statistical analysis was conducted using STATA 14.0 (StatCorp, College Station, USA). We categorized chocolate consumption into quintiles. The participants in the first quintile consumed no chocolate. Baseline characteristics were compared between the quintile groups of chocolate consumption using one-way analysis of variance (ANOVA) for continuous variables and chi-squared test for categorical variables. We excluded participants with prevalent baseline heart failure (where prevalent heart failure was defined using self-reported intake of drugs recommended for heart failure at the time of the survey, essentially loop diuretics in combination with digitalis or angiotensin converting enzyme inhibitors [22], [26]), cancer as well as participants with missing data on any of the variables included in the models. The bottom quintile (i.e. non-consumers) was used as the reference category for all risk estimates. Hazard ratios and corresponding 95% confidence intervals (95%CI) for the risk of incident heart failure were calculated using a Cox proportional hazards model.

The five different levels of adjustment for confounders are described in Appendix 2. In brief, cumulative models were constructed to control for potential lifestyle factors and co-morbid conditions (age, sex (model 1), lifestyle including dietary data: education, body mass index, social class, physical activity, smoking, dietary energy and alcohol consumption (model 2) and medical conditions that may contribute to the pathogenesis of heart failure: diabetes, myocardial infarction and arrhythmias (model 3)). We included other potential mediators (systolic blood pressure, cholesterol levels and heart rate [22]) in models 4a, b, and c, respectively. We then performed stratified analysis using model 3 for subgroups of participants based on: sex, age (<65 vs ≥65 years), body mass index (<25 vs ≥25 kg/m^2^), physical activity (active vs non-active), energy intake (<median energy vs ≥median energy), prior myocardial infarction, prior diabetes and prior arrhythmia. We formally tested for interaction by comparing the model with and without interaction term using the P-value of the likelihood ratio test (LRT). The interaction terms were presented graphically.

We also conducted a sensitivity analysis excluding participants who had incident heart failure within the first 3 years of the follow-up.

### Systematic review and meta-analysis

We aimed to identify studies that reported on the association between chocolate consumption and incident heart failure. We searched PubMed and EMBASE from inception until December 2013 using the terms (cocoa OR chocolate) AND (heart failure OR cardiac failure OR congestive cardiac failure OR left ventricular dysfunction), with no language restrictions and we checked bibliographies of included articles. We set up an automated search on PubMed for updates (most recent April 2015) on newly published studies with terms such as ‘chocolate’ and ‘heart or cardiac or cardiovascular’. We also performed a search of ESC 365 for unpublished studies. Two reviewers independently screened abstracts and titles, and then obtained full-text versions of potentially relevant studies to confirm eligibility. Studies were included if they evaluated chocolate intake and incident heart failure. Data extraction of included studies was performed by CSK and this was checked by YKL. We pooled data using the inverse variance method and random effects model in RevMan 5.2 software (Nordic Cochrane Center, Copenhagen, Denmark). For these comparisons, we considered subgroups of women, men, both genders combined and all studies irrespective of gender. We used the multivariable adjusted measures of association (hazard ratios, relative risks or odds ratios) for the highest category of chocolate consumption versus the lowest category of consumption. Heterogeneity was estimated using I^2^ where values greater than 50% were considered to have substantial heterogeneity [27]. In the case where a pooled estimate from meta-analysis contained more than 10 studies and there was no evidence of significant heterogeneity, we intended to evaluate publication bias using asymmetry testing in accordance to the recommendations by Ioannidis et al. [28].

## Results

### EPIC-Norfolk study

After excluding participants with prevalent cancer and heart failure at the baseline, a total of 20,922 participants, 9738 men and 11,184 women were included in the study (Appendix 3). The cohort's mean age at baseline was 58.0 ± 9.0 years and 53% were women. The mean follow up was 12.5 ± 2.7 years (total person years 262,291 years) during which 1101 individuals developed incident heart failure. A total of 4236 (20.3%) participants consumed no chocolate (less than once per month) in the past year.

The characteristics of participants according to quintiles of daily chocolate consumption are shown in Table 1. The highest consumers of chocolate consumed more than 15.5 g/d of chocolate while the participants in the bottom group consumed no chocolate. Higher chocolate consumption was significantly associated with a younger age and a lower systolic blood pressure, as well as a lower proportion of people with hypertension, myocardial infarction and diabetes, but associated with higher energy intake. The overall rates of incident heart failure were lower with higher chocolate consumption; crude rates were 3.3 events/1000 years and 5.7 events/1000 years in the highest and the lowest categories, respectively.

### Association between chocolate consumption and incident heart failure in the EPIC-Norfolk cohort

Table 2 shows that the highest chocolate consumption category was associated with lower risk estimates of incident heart failure in the age and sex adjusted model (HR 0.75 95%CI 0.62–0.91). However after adjustment for lifestyle factors and potential causal chronic co-morbidities, there was no significant difference between the highest consumers and no consumption of chocolate (HR 0.87 95%CI 0.71–1.06, Model 2). Further adjustments for all factors in a single model (Model 3), did not attenuate the results further and showed similar non-significant results (HR 0.87 95%CI 0.71–1.06).

Secondary analyses were conducted considering subgroups of participants (Table 3). The highest consumers of chocolate were associated with a lower risk of heart failure in the subgroup of participants with energy intake below the median (HR 0.68 95%CI 0.47–0.97). Models including an interaction term did not show significant improvement compared to model 3 in the prediction of incident heart failure (Appendix 4). In sensitivity analysis, we excluded participants with incident heart failure during the first 3 years (n = 88) and observed similar results (data not shown).

### Systematic review & meta-analysis

**Figure 1:**
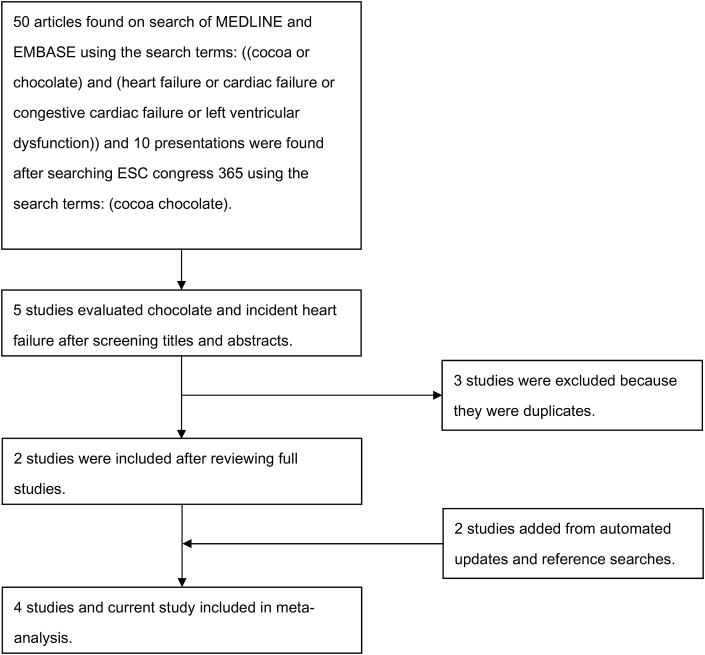
Search strategy and study selection.

Our search yielded 50 potentially relevant articles and 10 conference presentations. From these results, 4 studies met the inclusion criteria (Fig. 1). Details of the included studies are shown in Table 4. Two of the four studies were observational [15], [17] and two were post-hoc analysis of randomized trials [16], [18]. Two studies only included women [15], [16] one of which was conducted in women who underwent mammography [15], and the other one was conducted in women who were randomized to calcium therapy [16], one included only male physicians [18]. The remaining study included participants after myocardial infarction [17]. One study excluded participants with previous myocardial infarction [15]. Incident heart failure outcomes were standardized using International Classification of Disease codes in three studies.

All four studies used questionnaires to ascertain chocolate consumption, but there was heterogeneity in the way chocolate consumption was analyzed as one study considered frequency of chocolate consumption (monthly, weekly, two or more times weekly) [17], another compared one or more servings a week [16] and the others compared different number of servings (1–3 servings/month, 1–2 servings/week, 3–6 servings/week, ≥1 serving/day) [15], [18]. While all studies made some adjustments for confounders, age was adjusted for in all studies but smoking, exercise/physical activity and alcohol consumption were adjusted in three studies only [15], [17], [18].

**Figure 2:**
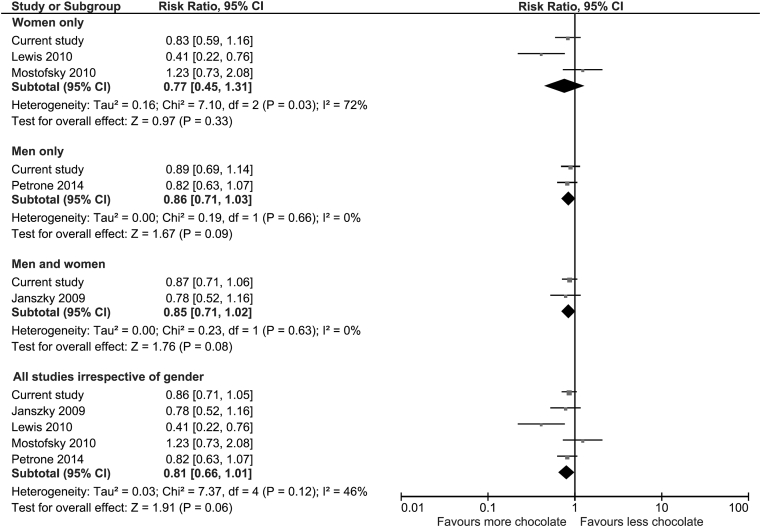
Meta-analysis of chocolate consumption and incident heart failure.

The pooled results of all the studies suggest that overall chocolate consumption was associated with a 19% relative risk reduction in incident heart failure but did not reach statistical significance (RR 0.81 95%CI 0.66–1.01, I^2^ = 46%) (Fig. 2). Considering the subgroups in Fig. 1 there were similar non-significant reductions in incident heart failure.

## Discussion

In this large prospective population based cohort study, systematic review and meta-analysis, we found the association between chocolate consumption and reduced subsequent risk of heart failure but this was not sufficiently robust after adjusting for potential confounders. This applied to men and women as well as those with or without baseline heart failure risk factors such as myocardial infarction, diabetes and arrhythmias. Considering these results in the context of existing studies, there appears to be consistent findings across all studies that chocolate consumption does not increase the risk of incident heart failure. We observed modest heterogeneity across studies and no significant association.

The EPIC-Norfolk cohort represents an apparently healthy middle and older age community dwelling population and thus the current study addresses previous limitations such as data from specific patient populations, or focused on a particular sex with certain characteristics and risk profile [15], [16], [17], [18].

Previous mechanistic studies have suggested that chocolate consumption could lower the risk of incident heart failure through several pathways. Cocoa is rich in flavonoids which are believed to be responsible for the cardioprotective properties of chocolate. Flammer et al. randomized participants with heart failure to flavanol-rich chocolate or placebo and found that chocolate led to peripheral vasodilatation, improvement in endothelial function and inhibition of platelet function [29]. However, their study was limited due to small sample size and thus no conclusive evidence was drawn for other outcomes such as blood pressure and measures of oxidative stress. A meta-analysis of ten randomized controlled trials has found that flavanol-rich cocoa products have the capacity to lower blood pressure [1]. This blood pressure reduction was thought to be due to flavanol inhibition of angiotensin converting enzyme [5]. Given the fact that ACE inhibitors are the cornerstone medical therapy for heart failure, this effect may be relevant in the observed association with heart failure incidence. In addition, a small randomized study has suggested that cocoa powder and dark chocolate consumption may modestly reduce LDL oxidation susceptibility, increase serum total antioxidant capacity and HDL cholesterol concentrations [30].

There is also evidence to suggest that chocolate has anti-inflammatory [13] and antiplatelet properties [31], [32]. Studies of dark chocolate have further provided evidence that consumption of chocolate improves endothelial function [33], increases levels of HDL cholesterol concentration [11] and lower levels of inflammation in the serum [12]. However, a recent study investigating the impact of dietary flavan-3-ol intake in the EPIC-Norfolk cohort suggests that the dose is insufficient to confer cardiovascular benefit so other mechanisms must account for the current findings [34]. Although we found trends towards non-significant relative risk reduction, reverse causality remains the main possible explanation. We observed that among lower categories of chocolate consumption there were more people with hypertension, previous myocardial infarction and diabetes. It may be that these participants with cardiovascular risk factors consumed less chocolate.

It should be noted that the chocolate consumption in the EPIC-Norfolk was skewed (the median consumption (IQR) was 4.6 (0.56–12)) and we also truncated chocolate consumption at max of 100 g/d to exclude outliers. We thus further examined the effect of incremental increase in chocolate consumption and incident heart failure and there was no evidence for harm (data not shown). We have recently reported that chocolate consumption was associated with favorable cardiovascular outcomes [14]. What remains controversial is that while the benefits of chocolate appear to be in those in the top quintiles of chocolate consumption, commercially available chocolate has high calorie content thus when consumed in high quantity may have adverse consequences because of increased weight gain [35], [36] and diabetes [37]. Therefore, it is possible that in some people the potential benefits of chocolate outweigh the consequences of increased caloric intake with resultant adverse effects such as poor diabetes control leading to complications.

While all studies used food frequency questionnaires to estimate chocolate consumption, we note that chocolate intake had no consistent definition across the studies. Most of the studies considered chocolate from chocolate bars while the study by Lewis et al. considered only solid chocolate and chocolate containing beverages [16]. In the current study, chocolate intake was derived from chocolate singles, chocolate bars and chocolate beverages. There was additional heterogeneity in the way the chocolate intake was categorized. Lewis et al. categorized chocolate intake from <1 serving/week to ≥7 servings/week [16]. In the study by Mostofsky et al. [15], there were 8 predefined responses ranging from never to ≥3 times per day for chocolate intake and Janszky used similar definitions [17]. Petrone et al. used frequency across the year from never or less than once per month to up to 5 times weekly [18]. In the current study, we analyzed the data according to quintiles of chocolate intake.

We observed that among the highest consumers of chocolate who had energy intake below the median had a lower risk of heart failure. It is unclear why this is. One possible reason for this observation may be that low energy intake may reflect individuals who are of younger age and younger age is associated with lower heart failure risk.

While we found that chocolate intake may possibly be associated with lower likelihood of heart failure, the association was not statistically significant after adjustments for potential confounders and mediators. There could still be a significant association but this study may not have had sufficient sample size and power to detect this. This is supported by the meta-analysis which pooled existing studies which all seem to suggest a lower risk in heart failure with higher chocolate consumption. However, the positive associations from published literature may also stem from publication bias whereby studies that show significant associations are more likely to be published whereas those with null or non-significant findings remain unpublished. More studies with high quality methods and adequately powered sample size are needed to determine if there are benefits of chocolate in terms of heart failure risk.

Our study has several strengths. The current study is based on a large population-based cohort thus is applicable to the middle and older aged general population. The characteristics of the EPIC-Norfolk participants were comparable to other UK representative samples except with lower prevalence of current smokers [18]. The sample size was sufficiently large to capture a sufficient number of incident heart failure events. Furthermore, we were able to account for many potential confounders including, socio-demographic, lifestyle, physiological, co-morbidity and dietary factors, which increased the robustness of our findings. In addition, clinically significant incident heart failure cases were adequately ascertained from hospital admissions [38] and death certificates. Furthermore, we conducted a systematic review to evaluate how our results compare with the existing literature and able to quantify the evidence by conducting a meta-analysis of similar studies.

Our study has some limitations. The FFQ has limitations associated with it, such as assumptions about portion size, possible errors in completing the questionnaire due to memory and extended recall time of 1 year. Some participants may under-report especially with chocolate consumption. This could mean that the high chocolate consumers are people with more ‘reliable chocolate intakes’ and possibly with other positive behaviors or characteristics which lower risk of a cardiac outcome. We were unable to differentiate plain, milk and dark chocolate in this study and therefore unable to determine the effect of the different types of chocolate. Mild cases of heart failure might have been missed at baseline. However, sensitivity analysis excluding those diagnosed with heart failure within the first 3 years of follow up did not alter the results. Studies have shown that many patients with left ventricular systolic dysfunction are asymptomatic, undetected and untreated [39], [40]. However, the use of hospital records would identify the most clinically relevant cases of heart failure. The evaluation of chocolate across all studies included in our systematic review lacked consistency defining high or low intake and also varied in ascertaining chocolate consumption which introduced modestly high heterogeneity in the meta-analysis.

It is worth noting that while chocolate consumption may be associated with potential cardiovascular benefit with regard to incidence of stroke and ischemic cardiovascular disease; the different pathophysiological process which underpins the development and progression of heart failure is very different from ischemic pathology. The initial inverse association between higher chocolate consumption and incident heart failure was attenuated by adjustment of confounders and potential mediators. Nevertheless, we observed an unlikely increased harm from higher chocolate consumption with regard to the risk of developing subsequent heart failure in this general population.

## Contributors

PKM, AAW and CSK conceptualized and designed the study. RNL was responsible for data management and CSK analyzed the data and drafted the first draft of the manuscript. CSK and YKL led the systematic review with input from AAW and PKM in study protocol development. KTK and NJW are the Principal Investigators of EPIC-Norfolk cohort. All authors contributed to the study design and writing of the paper. PKM is the guarantor.

## Funding

The EPIC-Norfolk study was supported by grants from the Medical Research Council and Cancer Research UK.

## Disclosures

The authors report no relationships that could be construed as a conflict of interest.

## Figures and Tables

**Figure:**
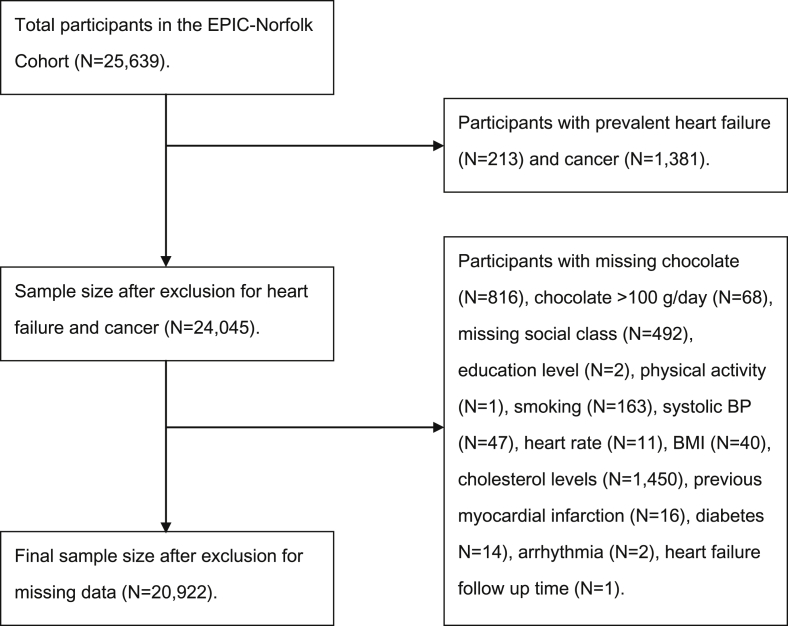


**Figure:**
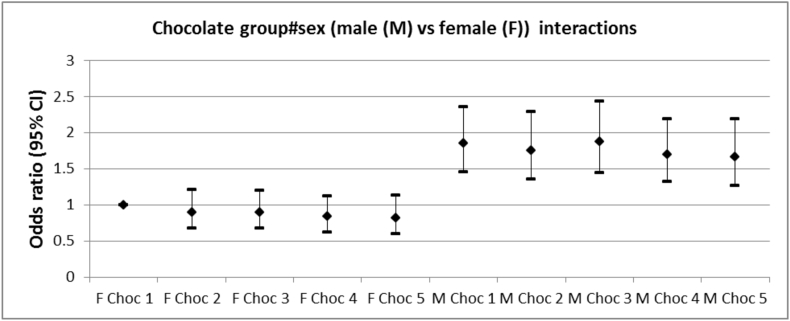


**Figure:**
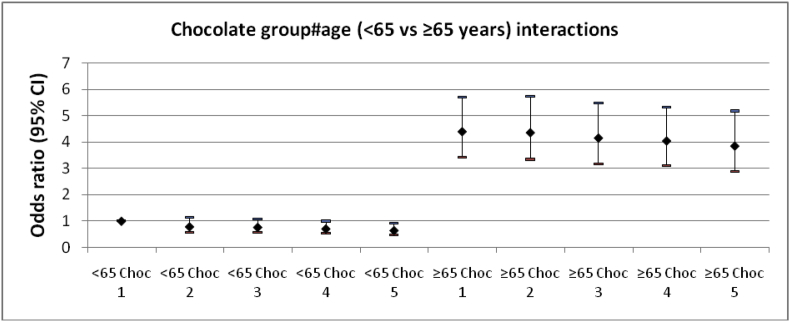


**Figure:**
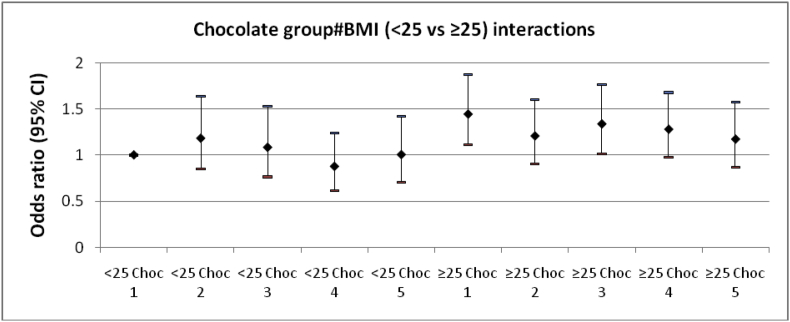


**Figure:**
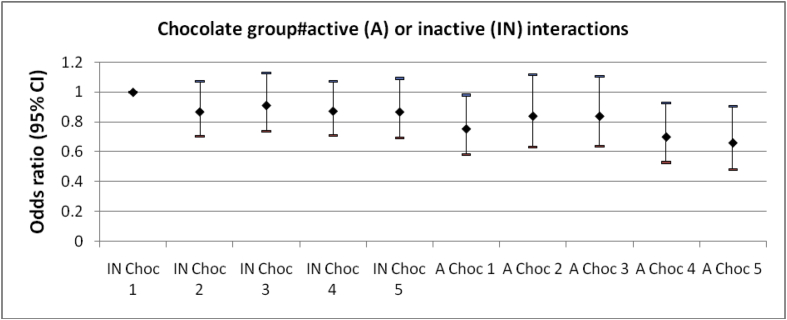


**Figure:**
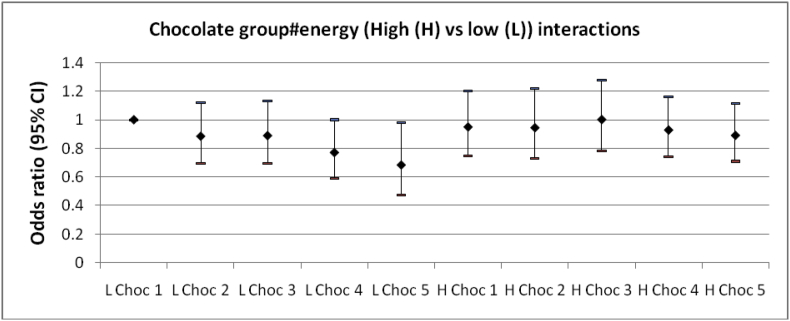


**Table 1:** Sample characteristics by chocolate intake of 20,922 men and women of the EPIC-Norfolk cohort at the study baseline (1993–1997) and incident heart failure at respective follow up March 2009.

Quintiles of daily chocolate intake	Quintile 1 (n = 4236)	Quintile 2 (n = 3521)	Quintile 3 (n = 4386)	Quintile 4 (n = 4582)	Quintile 5 (n = 4198)	p-value*
Range (g/day)	0	0.6–3.4	3.5–6.9	7.0–15.5	15.6–98.8	
**Median (IQR) (g/day)**						
Chocolate intake	0	0.8 (0.6, 1.4)	4.1 (4.1, 4.9)	8.7 (8.0, 12.0)	24.9 (22.1, 39.5)	
Chocolate singles	0	0.6 (0.6,1.1)	0.6 (0, 1.1)	1.1 (0, 3.4)	1.1 (0.6, 3.4)	
Chocolate bars	0	0	3.5 (3.5, 3.5)	7.0 (3.5, 7.0)	21.5 (21.5, 21.5)	
Chocolate beverages	0	0 (0, 0.8)	0 (0, 0.8)	0 (0, 5.2)	0 (0, 5.2)	
Sex, women	53 (2236)	56 (1962)	56 (2441)	54 (2455)	50 (2090)	<0.001
Age, years	60 ± 9	60 ± 9	57 ± 9	58 ± 9	57 ± 9	<0.001
Body mass index, kg/m^2^	26.4 ± 4.0	26.2 ± 3.8	26.3 ± 3.6	26.2 ± 3.8	26.1 ± 3.7	<0.001
Current smoker	10 (428)	10 (369)	11 (486)	12 (547)	13 (564)	<0.001
Self-reported diabetes	6 (235)	2 (60)	1 (60)	1 (47)	1 (31)	<0.001
Self-reported hypertension	16 (691)	14 (494)	13 (590)	13 (587)	12 (495)	<0.001
Self-reported myocardial infarction	4 (168)	3 (108)	2 (108)	2 (114)	2 (92)	<0.001
Self-reported arrhythmia	6 (239)	6 (194)	4 (190)	5 (225)	5 (213)	0.050
Total cholesterol, mmol/L	6.2 ± 1.2	6.2 ± 1.1	6.1 ± 1.1	6.2 ± 1.2	6.1 ± 1.2	<0.001
Systolic blood pressure, mmHg	137 ± 18	136 ± 18	135 ± 18	135 ± 18	134 ± 17	<0.001
Heart rate, beats/minute	71 ± 12	71 ± 12	70 ± 12	71 ± 12	71 ± 11	0.79
**Social class**						<0.001
I	6 (268)	8 (285)	7 (318)	7 (333)	6 (271)
II	34 (1429)	41 (1433)	39 (1698)	35 (1630)	36 (1496)
III non-manual	16 (685)	17 (590)	16 (692)	16 (754)	17 (739)
III manual	25 (1041)	20 (712)	23 (1008)	23 (1078)	24 (996)
IV	15 (630)	11 (398)	13 (562)	13 (618)	13 (556)
V	4 (183)	3 (103)	2 (108)	4 (168)	3 (140)
**Education**						<0.001
No qualification	40 (1702)	34 (1181)	34 (1499)	35 (1616)	34 (1445)	
O-level	10 (420)	10 (365)	11 (464)	11 (514)	11 (448)	
A-level	40 (1682)	41 (1440)	41 (1799)	41 (1867)	43 (1784)	
Degree or higher	10 (432)	15 (535)	14 (624)	13 (584)	12 (521)	
**Physical activity**						<0.001
Inactive	34 (1420)	29 (1037)	27 (1196)	27 (1256)	28 (1158)
Moderately inactive	27 (1153)	30 (1070)	29 (1283)	30 (1375)	28 (1167)
Moderately active	21 (907)	23 (818)	24 (1057)	23 (1048)	24 (1023)
Active	18 (756)	17 (596)	19 (850)	20 (902)	20 (850)
Energy intake by FFQ, kJ/day	7812 ± 2291	8104 ± 2265	8339 ± 2309	8928 ± 2411	9926 ± 2666	<0.001
Alcohol by FFQ, g/day	8.8 ± 14.4	9.3 ± 13.0	9.2 ± 13.1	8.3 ± 12.0	8.4 ± 12.2	<0.001
Incident heart failure	7 (302)	6 (201)	5 (206)	5 (221)	4 (171)	<0.001

Data are presented as mean ± SD, percentage (number) or median (IQR).

*P-value determined by analysis of variance (ANOVA) for continuous variables and χ^2^ test for categorical variables for differences among groups.

**Table 2:** Hazard Ratios for incident heart failure outcome in 20,922 men and women of the EPIC-Norfolk cohort by chocolate consumption.

Quintiles of daily chocolate intake	Quintile 1 0 g/day (n = 4236)	Quintile 2 0.6–3.4 g/day (n = 3521)	Quintile 3 3.5–6.9 g/day (n = 4386)	Quintile 4 7.0–15.5 g/day (n = 4582)	Quintile 5 15.6–98.8 g/day (n = 4198)	p-value for trend across median chocolate intake in each group
Model 1	1.00 (ref)	0.83 (0.69–0.99)	0.87 (0.73–1.03)	0.78 (0.66–0.93)	0.75 (0.62–0.91)	0.013
Model 2	1.00 (ref)	0.93 (0.78–1.11)	0.97 (0.81–1.16)	0.89 (0.74–1.06)	0.87 (0.71–1.06)	0.193
Model 3	1.00 (ref)	0.92 (0.77–1.11)	0.96 (0.80–1.15)	0.89 (0.74–1.06)	0.87 (0.71–1.06)	0.194

Model 1: Age, sex adjusted.

Model 2: Age, sex, education, body mass index (per unit), social class, physical activity, smoking status, dietary energy (per kJ/day), alcohol consumption (per g/day), myocardial infarction, diabetes, arrhythmia adjusted.

Model 3: Age, sex, education, body mass index (per unit), social class, physical activity, smoking status, dietary energy (per kJ/day), alcohol consumption (per g/day), myocardial infarction, diabetes, arrhythmia, systolic blood pressure (per mmHg), cholesterol level (per mmol/L), heart rate (per beat) adjusted.

**Table 3:** Subgroup analysis with hazard ratios for incident heart failure outcome in 20,922 men and women of the EPIC-Norfolk cohort by chocolate consumption.

Quintiles of daily chocolate intake	Quintile 1 0 g/day	Quintile 2 0.6–3.4 g/day	Quintile 3 3.5–6.9 g/day	Quintile 4 7.0–15.5 g/day	Quintile 5 15.6–98.8 g/day	Likelihood -ratio test p-value*
Female (n = 11,184)	1.00 (ref)	0.97 (0.72–1.31)	0.96 (0.72–1.30)	0.89 (0.66–1.21)	0.83 (0.59–1.16)	0.97
Male (n = 9738)	1.00 (ref)	0.92 (0.73–1.16)	0.97 (0.78–1.22)	0.90 (0.72–1.13)	0.89 (0.69–1.14)
Age <65 years (n = 14,696)	1.00 (ref)	0.90 (0.63–1.29)	0.85 (0.62–1.18)	0.83 (0.59–1.16)	0.74 (0.52–1.05)	0.56
Age ≥65 years (n = 6226)	1.00 (ref)	0.96 (0.78–1.18)	0.93 (0.75–1.15)	0.89 (0.72–1.09)	0.82 (0.66–1.06)
BMI <25 km/m^2^ (n = 8379)	1.00 (ref)	1.26 (0.91–1.75)	1.14 (0.81–1.63)	0.95 (0.66–1.35)	1.09 (0.76–1.56)	0.38
BMI ≥25 km/m^2^ (n = 12,543)	1.00 (ref)	0.83 (0.67–1.03)	0.91 (0.74–1.13)	0.87 (0.71–1.07)	0.79 (0.62–1.00)
Inactive (n = 12,115)	1.00 (ref)	0.87 (0.70–1.08)	0.91 (0.74–1.13)	0.87 (0.70–1.07)	0.86 (0.68–1.09)	0.99
Active (n = 8807)	1.00 (ref)	1.12 (0.79–1.58)	1.12 (0.79–1.57)	0.95 (0.67–1.34)	0.91 (0.62–1.33)
Low energy intake (n = 10,457)	1.00 (ref)	0.89 (0.70–1.13)	0.90 (0.71–1.15)	0.76 (0.59–0.99)	0.68 (0.47–0.97)	0.58
High energy intake (n = 10,465)	1.00 (ref)	0.97 (0.74–1.29)	1.04 (0.79–1.35)	0.95 (0.74–1.22)	0.93 (0.72–1.20)
No prior MI (n = 20,332)	1.00 (ref)	0.96 (0.79–1.16)	0.98 (0.81–1.19)	0.91 (0.75–1.10)	0.88 (0.71–1.09)	0.98
Prior MI (n = 590)	1.00 (ref)	0.80 (0.47–1.34)	0.93 (0.58–1.49)	0.76 (0.46–1.28)	0.78 (0.43–1.42)
No prior diabetes (n = 20,489)	1.00 (ref)	0.95 (0.79–1.15)	1.01 (0.84–1.22)	0.91 (0.75–1.09)	0.90 (0.73–1.10)	0.55
Prior diabetes (n = 433)	1.00 (ref)	0.93 (0.49–1.77)	0.54 (0.25–1.20)	0.74 (0.34–1.61)	0.50 (0.15–1.65)
No prior arrhythmia (n = 19,861)	1.00 (ref)	0.96 (0.79–1.17)	1.03 (0.85–1.24)	0.92 (0.76–1.11)	0.89 (0.72–1.10)	0.45
Prior arrhythmia (n = 1061)	1.00 (ref)	0.69 (0.40–1.17)	0.55 (0.30–1.01)	0.81 (0.47–1.38)	0.76 (0.44–1.32)

Adjusted for age, sex, education, body mass index (per unit), social class, physical activity, smoking status, dietary energy (per kJ/day), alcohol consumption (per g/day), myocardial infarction, diabetes, and arrhythmia.

* P-value reflects the comparison of the models without and with the interaction term (Please note, the HR presented in the table are from the stratified analysis).

**Table 4:** Studies of chocolate and heart failure.

Study	Design, Country	Types of participants	Number of participants	Exposure ascertainment	Outcome ascertainment	Use of adjustments	Results
Janszky 2009 [17]	Cohort study, Sweden	Non-diabetic participants post acute myocardial infarction in Stockholm Heart Epidemiology Program.	1169	Self-reported usual chocolate consumption.	Congestive heart failure events based on ICD-9 and 10 codes.	Adjusted for age, sex, smoking, obesity, physical inactivity, alcohol use, coffee intake, education and sweet score.	Congestive heart failure with less than once per month chocolate adjusted HR 0.82 (0.56–1.19), up to once per week chocolate adjusted HR 0.68 (0.47–0.97), twice or more a week chocolate adjusted HR 0.78 (0.52–1.16) compared to never consumption of chocolate.
Lewis 2010 [16]	Post-hoc analysis of RCT, Australia	Older women randomized to calcium supplementation.	1216	Chocolate consumption using validated questionnaire.	Heart failure events based on ICD-10-AM codes.	Adjusted for age, body mass index, socioeconomic status and energy intake.	Chocolate serving/week ≥1 vs <1: event rate 18/637 (2.8%) vs 35/579 (6%); adjusted OR 0.41 0.22–0.76, p = 0.01.
Mostofsky 2010 [15]	Cohort study, Sweden	Middle-aged and elderly women in Swedish Mammography Cohort.	31,823	Chocolate consumption using food frequency questionnaire.	Heart failure events based on ICD-9 and 10 codes.	Adjusted for total energy, age, education, body mass index, physical activity, smoking, living alone, postmenopausal hormone use, alcohol consumption, family history, hypertension and high cholesterol.	Chocolate vs no chocolate: 1–3 serving/month HR 0.74 (0.58–0.95), 1–2 serving/week HR 0.68 (0.50–0.93), 3–6 servings/week HR 1.09 (0.74–1.62), ≥1 serving/day HR 1.23 (0.73–2.08).
Petrone 2014 [18]	Post-hoc analysis of RCT, USA.	US male physicians who were randomized to low-dose aspirin. β-carotene, vitamin C, E and multivitamin in the Physicians' Health Study.	20,278	Chocolate consumption using food frequency questionnaire.	Heart failure based on annual follow-up questionnaires mailed to each participant and diagnoses were previously validated by reviewing medical records in a subsample.	Adjusted for age, BMI, alcohol consumption, smoking, exercise, caloric intake and prevalent atrial fibrillation.	Chocolate intake frequency and heart failure (Model 1): Never or <1/month: HR 1.00 1–3/month: HR 0.86 (0.72–1.03) 1/week: HR 0.80 (0.66–0.98) 2–4/week: HR 0.92 (0.74–1.13) 5+/week: HR 0.82 (0.63–1.07)
Current study	Cohort study, United Kingdom	General population.	20,987	Chocolate consumption based on food frequency questionnaire.	Incident heart failure events based on linkage to admissions database.	Adjusted for age, education level, social class, physical activity, smoking status, body mass index, myocardial infarction, diabetes, arrhythmia, dietary energy and alcohol consumption.	Chocolate consumption in highest vs lowest quintile: entire cohort adjusted HR 0.85 95%CI 0.71–1.05. Subgroup of women adjusted HR 0.81 (0.58–1.13) and subgroup of men adjusted HR 0.89 (0.69–1.14).

## Appendices

### Appendix 1 Description of data collection

Education status was recorded as degree or equivalent, A-level or equivalent, O-level or equivalent, and less than O-level or no qualifications [1]. Social class was classified according to the Registrar General's occupation-based classification scheme where social class I consists of professionals (e.g. Doctors and lawyers), class II includes managerial and technical occupations, class III is subdivided into non-manual and manual skilled workers (III non-manual and III manual), class IV consist of partly skilled workers, and class V comprises of unskilled manual workers [1]. Physical activity was assessed with two questions referring to usual physical activity at work, and physical activity outside work, during the past year [1]. The responses to these questions were used to allocate participants as: inactive (sedentary job and no recreational activity), moderately inactive (sedentary job with <0.5 h recreational activity per day or standing job with no recreational activity), moderately active (sedentary job with 0.5–1 h recreational activity per day or standing job with <0.5 h recreational activity per day or physical job with no recreational activity); and active (sedentary job with >1 h recreational activity per day or standing job with >1 h recreational activity per day or physical job with at least some recreational activity or heavy manual job).

Smoking history was obtained from yes–no responses to the questions: “Have you ever smoked as much as one cigarette a day for as long as a year?” and “Do you smoke cigarettes now?” and the responses were used to categorize participants as never or former smokers and current smokers [2]. Trained nurses examined each participant at a clinic visit. Height and weight were measured, and body mass index (BMI) was estimated as weight (kg) divided by height squared (m^2^) [3]. Blood pressure and heart rate were measured twice with an Accutorr non-invasive blood pressure monitor (Datascope Medical, Huntington, UK) after the participant had been seated for 5 min and the mean of two measurements was used for the analysis [3].

Blood samples were obtained using standard protocols and plasma was stored at −70 °C before total cholesterol concentrations were measured by colorimetry (RA 1000; Bayer Diagnostics, Basingstoke, UK) [1]. LDL cholesterol was calculated by the Friedewald formula. Participants were asked the question, “Has the doctor ever told you that you have any of the following?” and this was followed by a checklist to include myocardial infarction, cancer, diabetes and arrhythmia in order to identify known co-morbidities [1].

A food frequency questionnaire (FFQ) was used to assess overall diet in the past year. Food items in the questionnaire had nine answer categories: never or less than once per month, 1–3 per month, once a week, 2–4 per week, 5–6 per week, once a day, 2–3 per day, 4–5 per day, 6+ per day. This instrument and the way it is analyzed using the Compositional Analyses from Frequency Estimates (CAFÉ) and FFQ EPIC Tool for Analysis (FETA), are described in detail elsewhere [4], [5]. Alcohol consumption was derived from a questionnaire containing selected foods collected at the baseline clinic visit and the average alcohol consumption in grams/day was calculated [1].

**References**

1. McFadden E, Luben R, Wareham N, Bingham S, Khaw KT. Social class, risk factors and stroke incidence in men and women: a prospective study in the European Prospective Investigation into Cancer in Norfolk Cohort. Stroke 2009; 40:1070–1077.
2. Canoy D, Wareham N, Luben R, Welch A, Bingham S, Day N, Khaw KT. Cigarette smoking and fat distribution in 21,828 British men and women: a population-based study. Obesity Research 2005; 13: 1466–1475.
3. Pfister R, Michels G, Sharp SJ, Luben R, Wareham NJ, Khaw KT. Resting heart rate and incident heart failure in apparently healthy men and women in the EPIC-Norfolk study. Eur J Heart Fail 2012; 14:1163–70.
4. Welch AA, Luben R, Khaw KT, Bingham SA. The CAFE computer program for nutritional analysis of the EPIC-Norfolk food frequency questionnaire and identification of extreme nutrient values. Journal of Human Nutrition and Diet 2005; 18: 99–116.
5. Mulligan AA, Luben RN, Bhaniani A, Parry-Smith DJ, O'Connor L, Khawaja AP, Khaw KT. A new tool for converting food frequency questionnaire data into nutrient and food group values: FETA research methods and availability. BMJ Open 2014; 4:e004503.

### Appendix 2 Description of adjustments

Regression model 1 adjusted for age and sex only. Regression model 2 adjusted for age, education (4 categories: degree or equivalent, A-level or equivalent, O-level or equivalent, and less than O-level or no qualifications), social class (6 categories: class I consists of professionals, class II includes managerial and technical occupations, class III is subdivided into non-manual and manual skilled workers (III non-manual and III manual), class IV consist of partly skilled workers, and class V comprises of unskilled manual workers), physical activity (4 categories: inactive, moderately inactive, moderately active and active), dietary energy (kJ/day), body mass index (per unit), alcohol consumption (g/day) and current smoking which are important potential confounders with regard to habitual chocolate consumption. Regression model 3 adjusted for same variables as model 2 with the addition of diabetes, previous myocardial infarction and arrhythmia. Regression model 4 adjusted for all variables in model 3 but included the mediator systolic blood pressure (per mmHg) in 4a, serum cholesterol levels (per mmol/L) in 4b and pulse rate (per beat) in 4c. Regression model 5 is the complete model which adjusted for all factors in models 2–4.

### Appendix 4 Interaction terms for subgroup analysis of chocolate and risk of incident heart failure

A) Gender interactions

B) Age interactions

C) BMI interactions

D) Physical activity interactions

E) Energy intake interactions
